# Association Analysis of Polymorphic Variants of the *BDNF* Gene in Athletes

**DOI:** 10.3390/genes12091340

**Published:** 2021-08-28

**Authors:** Marta Niewczas, Paweł Król, Wojciech Czarny, Wojciech Bajorek, Marian Rzepko, Sławomir Drozd, Artur Płonka, Monika Drozd, Robert Czaja, Wiesław Błach, Pavel Ruzbarsky, Krzysztof Chmielowiec, Monika Michałowska-Sawczyn, Anna Grzywacz

**Affiliations:** 1 Faculty of Physical Education, University of Rzeszow, Towarnickiego 3 St., 35-959 Rzeszów, Poland; mniewczas@ur.edu.pl (M.N.); pkrol@ur.edu.pl (P.K.); wojciechczarny@wp.pl (W.C.); wbajorek@ur.edu.pl (W.B.); mrzepko@ur.edu.pl (M.R.); sdrozd@ur.edu.pl (S.D.); arplonka@ur.edu.pl (A.P.); mdrozd@ur.edu.pl (M.D.); rczaja@ur.edu.pl (R.C.); 2 Faculty of physical Education and Sport, School of Physical Education in Wrocław, Paderewskiego 3 St., 51-612 Wrocław, Poland; wieslaw.blach@awf.wroc.pl; 3 Faculty of Sports, University of Presov, St. 17. Novembra 15, 080 01 Prešov, Slovakia; pavel.ruzbarsky@unipo.sk; 4 Department of Hygiene and Epidemiology, Collegium Medicum, University of Zielona Góra, 28 Zyty St., 65-046 Zielona Góra, Poland; chmiele@vp.pl; 5 Faculty of Physical Education, Gdansk University of Physical Education and Sport, 1 K.Górskiego St., 80-336 Gdansk, Poland; monikamichalowska@op.pl; 6 Independent Laboratory of Health Promotion, Pomeranian Medical University in Szczecin, 11 Chlapowskiego St., 70-204 Szczecin, Poland

**Keywords:** *BDNF*, genes, athletes, personality, rs10767664, rs2030323

## Abstract

As *BDNF* is one of the group of neurotrophins highly influencing the processes happening in the brain, such as the processes of learning and personality creation, we decided to look closer at its genetic variations in association with the personality of a group of athletes and their controls. The study group consisted of 305 volunteers: martial arts athletes (*n* = 153; mean age = 24.06) and healthy non-athletes as controls (*n* = 152; mean age = 22.23). Thirty-eight percent of the martial arts group achieved the championship level. Both the martial arts and control subjects were examined using the NEO Five-Factor Personality Inventory (NEO-FFI) and the State-Trait Anxiety Inventory (STAI) scales. The results of the NEO-FFI and STAI inventories were given as sten scores. The conversion of the raw score to the sten scale was performed according to Polish norms for adults. Genomic DNA was extracted from blood leukocytes and then genotyped using a PCR method for the following polymorphisms: *BDNF* rs10767664 and *BDNF* rs2030323. We observed statistical significance for both polymorphisms when comparing martial arts athletes with the control group in relation to the conscientiousness and extraversion scales. However, since few extant articles consider this association, our results still require further analysis, probably by considering a larger group.

## 1. Introduction

Brain-derived neurotrophic factor (*BDNF*) is one of the most considered neurotrophins due to its importance in homeostasis, health, and disease. However, being one of the most widely studied neurotrophins, numerous questions concerning BDNF’s connection to molecular biology and signaling pathways still remain. Although there is a lot of information concerning *BDNF*’s gene structure, peptide composition, signaling pathways, and the functional effects of genetic variations, the biology of *BDNF* is diverse and varied [1,2]. *BDNF* is, as a rule, expressed in both developing and adult mammalian brains where it is critical factor in neuronal survival, morphogenesis, brain plasticity, synaptic function, morphological change, and in the differences in neuronal response [3]. The mammalian nervous system presents highly experience-dependent changes in synapse structure and function and is considered an important element in memory formation [4], influencing the long-term potentiation (LTP) of hippocampal synapses, especially in the CA1 region. The sequence of plasticity formation is connected with some steps. The first step, known as early-LTP, is transcription- and translation-independent and lasts for 1–2 h; the second step, late-LTP, can last for hours or days and is connected to transcription and translation activity [5,6,7]. *BDNF* is considered a key mediation of activity-induced LTP in the hippocampus and other brain regions [8,9,10]. The modulatory effect of *BDNF* is connected to the modification of components already available at the synapse in case of early effects, while the long-term results from the modification of translation activity at the synapse changes in transcription. Such changes can be of high importance for behavioral plasticity that, as a result, modifies human personality [2].

In this aspect, changes in *BDNF* expression, release, and neuromodulatory activity can be mediated by both epigenetic and post-translational mechanisms. The association between many pathological conditions and developmental experiences has been emphasized in connection with plasticity in the hippocampus, which is traditionally considered an element in learning and memorizing processes; however, other important structures should also be considered, such as the amygdala, which is also induced by *BDNF* changes. Nonetheless, *BDNF* is shown to be a biochemical integrator of convergent cellular signals and a central element of neural plasticity [11,12,13].

Considering the importance of *BDNF* in learning processes and personality traits, we decided to concentrate on an association between physical activity, personality traits, and polymorphic differences. Some studies emphasize that environmental enrichment in a form of environmental variation [14], physical exercise [15], or social enrichment [16] can induce higher a level of BDNF in the brain, which in turn results in increased memorization and learning abilities [17]. Animal models also show an upregulation of *BDNF* gene expression, especially in the hippocampal area [18,19]; however, other structures of the nervous system were also influenced, among them the inferior temporal areas of the brain [20].

As all these areas and pathways are of high importance in the creation of personality, we considered it reasonable to search for an association between them. A direct relation exists between the brain-derived neurotrophic factor gene and personality traits. *BDNF* expression, being an important factor in learning processes, is influenced by genetic polymorphisms. One of the examples indicated on nucleotide polymorphism at nucleotide 196G/A, which results in the substitution of valine by methionine at codon 66 (val66met) of the pro-BDNF molecule, and therefore the presence of the met allele, was linked to the decreased activity-dependent secretion of *BDNF* [21]. However, the authors suggest that the existence of such an association has not been deeply investigated [22,23,24]. Taking this finding into consideration, we decided to direct our study to an investigation of the association between personality traits and polymorphisms in the *BDNF* gene.

## 2. Materials and Methods

### 2.1. Subjects

The study group consisted of 305 volunteers: martial arts (*n* = 153; mean age = 24.06; SD = 6.21; minimum 17.00; maximum 40.00; men 78%; women 22%; MMA, *n* = 28; judo, *n* = 25; boxing, *n* = 10; karate, *n* = 20; kickboxing, *n* = 13; ju-jitsu, *n* = 35; and wrestling, *n* = 22) and healthy non-athlete controls (*n* = 152; mean age = 22.23, SD = 4.55; minimum 17.00; maximum 50.00; men 85%; and women 15%). Thirty-eight percent of the martial arts group achieved the championship level. Informed, written consent was received from the participants of the study. The study was conducted according to the guidelines of the Declaration of Helsinki, and approved by KOMISJA BIOETYCZNA przy Uniwersytecie Rzeszowskim, ul. Warszawska 26A, 35-205 Rzeszów (protocol nr 3 November 2017, 9 November 2017).

The martial arts and control subjects were examined using the NEO Five-Factor Personality Inventory (NEO-FFI) and State-Trait Anxiety Inventory (STAI) scales.

The NEO Personality Inventory scale (NEO Five-Factor Inventory, NEO-FFI) was based on 6 dimensions for each of the five traits: extraversion (positive emotion, warmth, gregariousness, activity, excitement seeking, and assertiveness); agreeableness (tendermindedness, trust, altruism, straightforwardness, compliance, and modesty); openness to experience (fantasy, feelings, aesthetics, actions, values, and ideas); conscientiousness (deliberation, competence, dutifulness, order, achievement striving, and self-discipline); and neuroticism (anxiety, vulnerability to stress, hostility, self-consciousness, impulsiveness, and depression) [25].

Sten scores were used to present the results of the NEO-FFI and STAI inventories. The obtained raw scores were converted using the sten scale according to Polish norms for adults with the assumption that 1–2 indicated very low scores; 3–4 indicated low scores; 5–6 indicated average scores; 7–8 indicated high scores; 9–10 indicated very high scores.

### 2.2. Genotyping

Blood for the genetic assays was collected in tubes with EDTA (anticoagulant). Blood leukocytes were also used to obtain genomic DNA. We used a High Pure Polymerase Chain Reaction (PCR) Template Preparation Kit (Roche Diagnostics, Mannheim, Germany) to isolate the cell DNA. The process of extraction was conducted in accordance with the manufacturer’s instructions. The extracted DNA samples were stored at 4 °C until further analysis.

Venous blood collected according to standard procedures was the source of genomic DNA. The PCR method was used to genotype the samples. We used the set of Search TaqMan^®^ Assays from Thermo Fisher Scientific to identify the polymorphisms distribution.

### 2.3. Statistical Analysis

The concordance between the genotype frequency distribution and Hardy-Weinberg equilibrium (HWE) was checked with HWE software (https://wpcalc.com/en/equilibrium-hardy-weinberg/ (accessed on 3 June 2021)). The associations between *BDNF* rs10767664 and *BDNF* rs2030323, the martial arts and control subjects, and the NEO Five-Factor Inventory (NEO-FFI) were analyzed with a multivariate analysis of the factor effects of ANOVA (NEO-FFI/ scale STAI/ × genetic feature × control and martial arts × (genetic feature × control and martial arts)). The homogeneity of the variance condition was satisfied (Levene test *p* > 0.05). The normality of distribution was not fulfilled in the case of the analyzed variables. The NEO Five-Factor Inventory (neuroticism, extraversion, openness, agreeability, and conscientiousness) was analyzed and compared with the usage of the Mann–Whitney U test. A chi-square test was applied to compare genotype frequencies between healthy control subjects and martial arts athletes for *BDNF* rs10767664 and *BDNF* rs2030323 polymorphism. All calculations were performed using STATISTICA 13 (Tibco Software Inc, Palo Alto, CA, USA) for Windows (Microsoft Corporation, Redmond, WA, USA).

## 3. Results

The frequency distributions were in accordance with the HWE. No statistical difference was found between martial arts participants and people from the control group (Table 1).

The *BDNF* rs10767664 and *BDNF* rs2030323 genotypes and alleles frequencies in the studied sample did not differ in the analyzed subject groups (Table 2).

The means and standard deviations for the NEO Five-Factor Inventory results in the martial arts subject and control subject groups are presented in Table 3. In comparison with the controls, the case group subjects had significantly higher scores on the extraversion/scale (M 6.89 vs. M 6.43, *p* = 0.0405) and conscientiousness/scale (M 7.23 vs. M 5.89, *p* < 0.0001).

*Conscientiousness/scale and BDNF* rs10767664.

**Figure 1:**
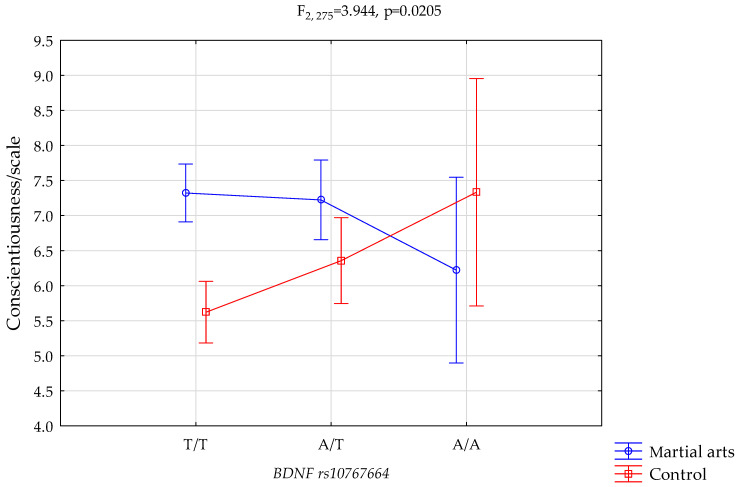
Interaction between s martial arts/control and *BDNF* rs10767664, and the conscientiousness scale.

The results of 2 × 3 factorial ANOVA illustrated a statistically significant effect of the combined factor *BDNF* rs10767664 genotype of martial arts/control (F_2275_ = 3.94, *p* = 0.0205, η^2^ = 0.028) (Table 4, Figure 1). Our sample had more than 71% power to detect the combined factor of martial arts/control × *BDNF rs10767664* and their interaction effect (about 2.8% of the phenotype variance). The post hoc analysis is shown in Table 4. The results of the post hoc test are included in Table 6.

*Conscientiousness/scale and BDNF* rs2030323.

**Figure 2:**
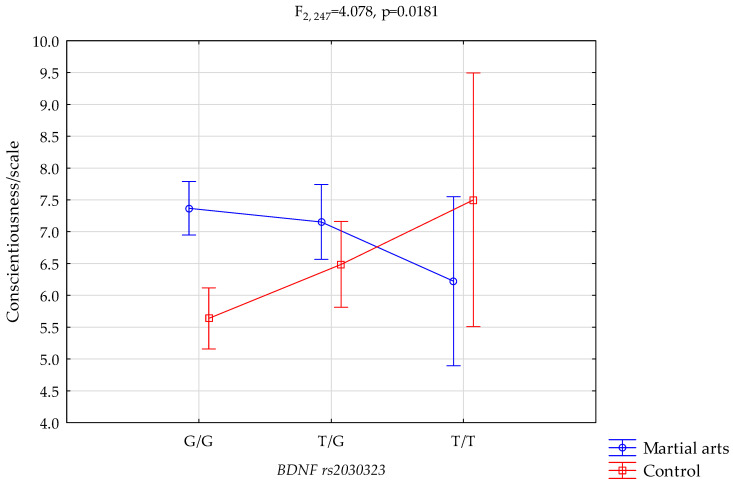
Interaction between s martial arts/control and *BDNF* rs2030323 and conscientiousness scale.

The results of 2 × 3 factorial ANOVA illustrated a statistically significant effect of the combined factor *BDNF* rs2030323 genotype of martial arts/control (F_2,247_ = 4.08, 0.0181, η^2^ = 0.032) (Table 5, Figure 2.). Our sample had more than 72% power to detect the combined factor of martial arts/control × *BDNF* rs2030323 and their interaction effect (about 3.2% of the phenotype variance). The post hoc analysis is shown in Table 4. The results of the post hoc test are included in Table 6.

## 4. Discussion

We observed a statistically significant interaction between the occurrence of T/T and A/T genotypes in *BDNF* rs10767664 among individuals practicing martial arts, and higher results on the NEO FFI sten and conscientiousness scales in comparison with the control group (Figure 1, 7.32 vs. 5.62, *p* = 0.0000; 7.22 vs. 6.35, *p* = 0.0420; Table 6).

We also noticed a statistically significant interaction between the frequency of G/G genotypes and higher results on the NEO FFI sten and conscientiousness scales by comparing the martial arts athletes with the control group (Figure 2, 7.37 vs. 5.64, *p* = 0.0000, Table 6).

The novelty of our scientific assumption is its combination of personality traits with genotype variants of BDNF polymorphism. This assumption is correct when we consider biological level due to the fact that BDNF influences brain neuroplasticity. Conscientiousness as a phenotype expressed with behavior might be conditioned with polymorphic variants of genes connected to neurotransmission [2].

As the level of BDNF is connected to different factors, amongst others, with physical activity, some research has concentrated on such an association. One study noticed the association between 35 min sessions of physical activity, cognitive training, and mindfulness practice in a group of healthy older adults. The results showed that a single bout of physical activity influenced serum BDNF levels more significantly than any of two other forms of activity. The authors emphasized that physical activity influenced serum BDNF by peripheral origin; however, the cognitive functions of an individual can be implied by it [26].

Humans, both younger and older, showed an association between their physical activity level and the BDNF level in the serum [27], even after a singular bout of training [28,29]. The authors noticed that barely any research exists concerning physical activity in their capacity to alter BDNF levels. Nonetheless, they assumed that physical exercises can show immediate effects on the serum levels of BDNF in healthy humans. What is interesting is that they also noticed that mindfulness practice can induce the same reaction as an increased level of BDNF. Few previously conducted longitudinal studies have suggested that that there is an association between meditation practice and cognition [30], and BDNF is suggested to be a key mediator in this situation [31]. Moreover, a combination of the two over a 12-week period improved visuospatial memory, which is probably connected to parallel changes in brain connectivity. Therefore, we can think about both physical activity and mindfulness training as elements with a high potential to change BDNF levels [32].

Polymorphisms in *BDNF* were found to be a modifying factor in cases of depression in children [33], as well as in incidents of depression in elders [34]. The authors of that research emphasized that BDNF val66met polymorphism did not directly influence ones’ vulnerability to depressive disorders, but that its occurrence was more probable following stressful events. The authors also indicated that such a relation exists not only between depression and *BDNF* polymorphism, but also between other health disorders, such as stroke, cancer, and subsequent depressive states [35,36]. Both these studies and our observations have underlined the fact that *BDNF* polymorphisms have the potential to influences health environments with long-term effects. However, the authors’ observations suggest that the results may differ for various populations [37,38].

Nevertheless, we should also mention some limitations of our research. In our research, we chose only to analyze BDNF; however, we are aware that the phenotypic manifestation of personality traits may also be influenced by other polymorphisms, as it is a polygenic and multifactorial feature. However, an analysis of BDNF is crucial and necessary to understand its biological bases. Nonetheless, further analyses concerning more numerous groups of athletes and higher number of genes are still needed. It also seems justified to analyze methylation in the promotor region of chosen genes.

## 5. Conclusions

Since we observed the statistical significance of both polymorphisms when comparing martial arts athletes with the control group in relation to the conscientiousness scale, we contend that it is an element of high importance in the creation of human personality. However, since few extant articles consider this association, our results still require further analysis, probably by considering a more numerous group.

## Figures and Tables

**Table 1:** Hardy-Weinberg equilibrium of *BDNF* rs10767664 and *BDNF* rs2030323 in the groups of martial arts subjects and controls.

	Martial Arts *n* = 151 Observed (Expected)	χ^2^ (*p* Value) Alleles Frequency	Controls *n* = 130 Observed (Expected)	χ^2^ (*p* Value) Alleles Frequency
*BDNF* rs10767664				
T/T	93 (91.4)	0.546 (0.4599)	82 (81.6)	0.044 (0.8344)
A/T	49 (52.1)	p allele freq (T) = 0.78	42 (42.8)	p allele freq (T) = 0.79
A/A	9 (7.4)	q allele freq (A) = 0.22	6 (5.6)	q allele freq (A) = 0.21
*BDNF* rs2030323				
	*n =* 145		*n =* 108	
G/G	90 (88.1)	0.876 (0.3494)	69 (69.3)	0.029 (0.8658)
T/G	46 (49.9)	p allele freq (G) = 0.78	35 (34.4)	p allele freq (G) = 0.80
T/T	9 (7.1)	q allele freq (T) = 0.22	4 (4.3)	q allele freq (T) = 0.20

*p*—statistical significance, χ^2^—Chi^2^ test result, *n*—number of subjects.

**Table 2:** Frequency of genotypes and alleles of BDNF rs10767664 and *BDNF* rs2030323 in the groups of martial arts subjects and controls.

	Martial Arts	Controls	χ^2^ (*p* Value)
*BDNF* rs10767664			
	*n* = 151	*n* = 130	0.262 (0.8772)
T/T	93 (61.59%)	82 (63.08%)	
A/T	49 (32.45%)	42 (32.31%)	
A/A	9 (5.96%)	6 (4.62%)	
T	235 (77.81%)	206 (79.23%)	0.150 (0.7013)
A	67 (22.19%)	54 (20.77%)	
*BDNF* rs2030323			
	*n* = 145	*n* = 108	0.797 (0.6715)
G/G	90 (62.07%)	69 (63.89%)	
T/G	46 (31.72%)	35 (32.41%)	
T/T	9 (6.21%)	4 (3.70%)	
G	226 (77.93%)	173 (80.09%)	0.350 (0.5559)
T	64 (22.07%)	43 (19.91%)	

*p*—statistical significance, χ^2^—Chi^2^ test result, *n*—number of subjects.

**Table 3:** Analysis of NEO Five-Factor Inventory results in martial arts subjects and in controls.

STAI/NEO Five Factor Inventory/	Martial Arts (*n* = 153) M ± SD	Control (*n* = 152) M ± SD	U Mann-Whitney Z	*p* Value
Neuroticism/scale	4.76 ± 2.24	4.65 ± 1.92	−0.027	0.9788
Extraversion/scale	6.89 ± 1.95	6.43 ± 1.85	2.048	0.0405 *
Openness/scale	4.84 ± 1.79	4.56 ± 1.55	1.641	0.1007
Agreeability/scale	5.77 ± 2.26	5.71 ± 2.06	0.310	0.7563
Conscientiousness/scale	7.23 ± 2.09	5.89 ± 1.99	5.653	0.0000 *

M—mean, SD—standard deviation, Mann–Whitney U Z-test. *—significant statistical differences.

**Table 4:** The results of 2 × 3 factorial ANOVA for martial arts subjects and controls, NEO Five-Factor Inventory scale, and *BDNF* rs10767664.

	*BDNF* rs10767664					2 × 3-Factor ANOVA				
NEO Five Factor Inventory	Martial Arts (*n* = 151) M ± SD	Control (*n* = 130) M ± SD	T/T (*n* = 175) M ± SD	A/T (*n* = 91) M ± SD	A/A (*n* = 15) M ± SD	Full Model F (*p* Value)	Factor	F (*p* Value)	ɳ^2^	Power (alfa = 0.05)
Neuroticism/scale	4.80 ± 2.24	4.56 ± 1.96	4.53 ± 2.10	4.98 ± 2.12	4.87 ± 2.10	F_5275_ = 0.849 *p* = 0.5161 R^2^ = 0.015	intercept	F_1275_ = 527.56 (*p* < 0.0001)	0.657	1.000
							Martial arts/control	F_1275_ = 0.95 (*p* = 0.3293)	0.003	0.164
							*BDNF* rs10767664	F_2275_ = 1.42 (*p* = 0.2437)	0.010	0.303
							Martial arts/control × *BDNF* rs10767664	F_2275_ = 0.27 (*p* = 0.7639)	0.002	0.092
Extraversion/scale	6.91 ± 1.95	6.47 ± 1.87	6.77 ± 1.91	6.56 ± 1.978	6.87 ± 1.73	F_5275_ = 1.087 *p* = 0.3678 R^2^ = 0.019	intercept	F_1275_ = 1284.11 (*p* < 0.0001)	0.823	1.000
							Martial arts/control	F_1275_ = 0.14 (*p* = 0.7077)	0.0005	0.066
							BDNF rs10767664	F_2275_ = 0.43 (*p* = 0.6472)	0.003	0.120
							Martial arts/control × *BDNF* rs10767664	F_2275_ = 0.51 (*p* = 0.6015)	0.004	0.133
Openness/scale	4.85 ± 1.80	4.55 ± 1.61	4.78 ± 1.71	4.57 ± 1.74	4.80 ± 1.70	F_5,275_ = 0.8121 *p* = 0.5419 R^2^ = 0.014	intercept	F_1275_ = 769.32 (*p* < 0.0001)	0.737	1.000
							Martial arts/control	F_1275_ = 2.28 (*p* = <0.1324)	0.008	0.324
							BDNF rs10767664	F_2275_ = 0.40 (*p* = 0.6691)	0.002	0.115
							Martial arts/control × *BDNF* rs10767664	F_2275_ = 0.46 (*p* = 0.6299)	0.003	0.125
Agreeability/scale	5.80 ± 2.25	5.86 ± 2.06	5.92 ± 2.24	5.69 ± 2.01	5.60 ± 2.20	F_5,275_ = 0.2357 *p* = 0.9465 R^2^ = 0.004	intercept	F_1275_ = 731.30 (*p* < 0.0001)	0.727	1.000
							Martial arts/control	F_1275_ = 0.32 (*p* = 0.5735)	0.001	0.087
							*BDNF* rs10767664	F_2275_ = 0.37 (*p* = 0.6928)	0.003	0.109
							Martial arts/control × *BDNF* rs10767664	F_2275_ = 0.15 (*p* = 0.8600)	0.001	0.073
Conscientiousness/scale	7.22 ± 2.10	5.94 ± 1.96	6.53 ± 2.24	6.82 ± 1.88	6.66 ± 2.32	F_5275_ = 7.5012 *p* = 0.0000 * R^2^ = 0.120	intercept	F_1275_ = 1142.88 (*p* < 0.0001 *)	0.806	1.000
							Martial arts/control	F_1275_ = 1.51 (*p* = 0.2202)	0.005	0.231
							*BDNF* rs10767664	F_2275_ = 0.81 (*p* = 0.4476)	0.006	0.187
							Martial arts/control × *BDNF* rs10767664	F_2275_ = 3.94 (*p* = 0.0205 *)	0.028	0.706

M—mean, SD—standard deviation. *—significant statistical differences.

**Table 5:** The results of 2 × 3 factorial ANOVA for martial arts subjects and controls, NEO Five Factor Inventory scale and *BDNF* rs2030323.

	*BDNF* rs2030323					2 × 3-Factor ANOVA				
NEO Five Factor Inventory	Martial Arts (*n* = 145) M ± SD	Control (*n* = 108) M ± SD	G/G (*n* = 159) M ± SD	T/G (*n* = 81) M ± SD	T/T (*n* = 13) M ± SD	Full Model F (*p* Value)	Factor	F (*p* Value)	ɳ^2^	Power (alfa = 0.05)
Neuroticism/scale	4.81 ± 2.24	4.54 ± 1.96	4.53 ± 2.09	4.99 ± 2.18	4.77 ± 2.17	F_5247_ = 0.9104 *p* = 0.4748 R^2^ = 0.018	intercept	F_1247_ = 395.10 (*p* < 0.0001)	0.615	1.000
							Martial arts/control	F_1247_ = 1.98 (*p* = 0.1607)	0.007	0.289
							*BDNF* rs203032*3*	F_2247_ = 1.14 (*p* = 0.3197)	0.009	0.251
							Martial arts/control × *BDNF* rs2030323	F_2247_ = 0.56 (*p* = 0.5720)	0.004	0.142
Extraversion/scale	6.93 ± 1.96	6.63 ± 1.87	6.87 ± 1.89	6.62 ± 2.02	7.15 ± 1.67	F_5247_ = 1.1199 *p* = 0.3502 R^2^ = 0.022	intercept	F_1247_ = 1082.83 (*p* < 0.0001)	0.814	1.000
							Martial arts/control	F_1247_ = 0.44 (*p* = 0.5067)	0.002	0.101
							*BDNF* rs2030323	F_2247_ = 1.14 (*p* = 0.3226)	0.009	0.249
							Martial arts/control × *BDNF* rs2030323	F_2247_ = 1.39 (*p* = 0.2501)	0.011	0.298
Openness/scale	4.89 ± 1.79	4.57 ± 1.52	4.79 ± 1.72	4.62 ± 1.66	5.08 ± 1.61	F_5247_ = 0.6666 *p* = 0.6491 R^2^ = 0.013	intercept	F_1247_ = 656.57 (*p* < 0.0001)	0.727	1.000
							Martial arts/control	F_1247_ = 0.88 (*p* = 0.3478)	0.004	0.155
							*BDNF* rs203032*3*	F_2247_ = 0.39 (*p* = 0.6737)	0.003	0.113
							Martial arts/control × *BDNF* rs2030323	F_2247_ = 0.04 (*p* = 0.9567)	0.0003	0.057
Agreeability/scale	5.86 ± 2.26	6.02 ± 1.93	6.04 ± 2.22	5.69 ± 1.96	6.00 ± 1.91	F_5247_ = 0.9234 *p* = 0.4662 R^2^ = 0.018	intercept	F_1247_ = 666.58 (*p* < 0.0001)	0.729	1.000
							Martial arts/control	F_1247_ = 2.46 (*p* = 0.1176)	0.010	0.346
							*BDNF* rs2030323	F_2247_ = 1.07 (*p* = 0.3426)	0.009	0.238
							Martial arts/control × *BDNF* rs2030323	F_2247_ = 1.39 (*p* = 0.2502)	0.011	0.298
Conscientious-ness/scale	7.23 ± 2.10	5.98 ± 1.97	6.62 ± 2.23	6.86 ± 1.87	6.61 ± 2.47	F_5247_ = 6.5157 *p* = 0.00001 * R^2^ = 0.117	intercept	F_1247_ = 909.86 (*p* < 0.0001 *)	**0.786**	**1.000**
							Martial arts/control	F_1,247_ = 0.70 (*p* = 0.4044)	0.003	0.132
							*BDNF* rs2030323	F_2247_ = 0.73 (*p* = 0.4843)	0.006	0.172
							Martial arts/control × *BDNF* rs2030323	F_2247_ = 4.08 (*p* = 0.0181 *)	0.032	0.721

M—mean, SD—standard deviation. *—significant statistical differences.

**Table 6:** Post hoc analysis of interactions between martial arts/control and *BDNF* rs10767664/*BDNF* rs2030323, and the conscientiousness scale.

*BDNF rs10767664* and NEO FFI Conscientiousness scale						
	{1} M = 7.32	{2} M = 7.22	{3} M = 6.22	{4} M = 5.62	{5} M = 6.35	{6} M = 7.33
Martial arts *BDNF* T/T {1}		0.7833	0.1196	0.0000 *	0.0106 *	0.9899
Martial arts *BDNF* A/T {2}			0.1721	0.0000 *	0.0420 *	0.9009
Martial arts *BDNF* A/A {3}				0.3978	0.8557	0.2972
Control *BDNF* T/T {4}					0.0560	0.0460 *
Control *BDNF* A/T {5}						0.2688
Control *BDNF* A/A {6}						
*BDNF rs2030323* and NEO FFI Conscientiousness scale						
	{1} M = 7.37	{2} M = 7.15	{3} M = 6.22	{4} M = 5.64	{5} M = 6.49	{6} M = 7.50
Martial arts *BDNF* G/G{1}		0.559326	0.107113	0.000000 *	0.029850 *	0.897538
Martial arts *BDNF* T/G {2}			0.208701	0.000110 *	0.143413	0.741958
Martial arts *BDNF* T/T {3}				0.415967	0.727921	0.294536
Control *BDNF* G/G {4}					0.044587 *	0.074854
Control *BDNF* T/G {5}						0.343362
Control *BDNF* T/T {6}						

*—significant statistical differences, M—mean.

## Data Availability

Not applicable.
